# Assessing the association between supplemented puppyhood dietary fat sources and owner-reported epilepsy in adulthood, among Finnish companion dogs

**DOI:** 10.3389/fvets.2023.1227437

**Published:** 2023-09-15

**Authors:** Manal Hemida, Sarah Rosendahl, Tarja S. Jokinen, Robin Moore, Kristiina A. Vuori, Johanna Anturaniemi, Anna Hielm-Björkman

**Affiliations:** ^1^Department of Equine and Small Animal Medicine, Faculty of Veterinary Medicine, University of Helsinki, Helsinki, Finland; ^2^Department of Nutrition and Clinical Nutrition, Faculty of Veterinary Medicine, Beni-Suef University, Beni Suef, Egypt

**Keywords:** epilepsy, dog, feed, fat, fish, epidemiology

## Abstract

**Introduction:**

Epilepsy is a serious and common neurological condition in dogs, despite the wide number of antiepileptic drugs available, in approximately one third of the patients, epilepsy remains unsatisfactorily controlled. We aim to analyze whether feeding dietary fat sources during puppyhood was associated with canine epilepsy in adulthood.

**Methods:**

A nested case–control study was compiled from the validated DogRisk food frequency questionnaire (DogRisk FFQ). DogRisk FFQ collected feeding, disease, and background data about the dog. The study sample consisted of 108 owner-reported epileptic cases and 397 non-epileptic controls. Each case was matched with up to four controls for the key confounding factors of sex, breed, and age. We analyzed associations between feeding as a puppy and owner-reported epilepsy as an adult dog using Cox regression. We tested 55 different food variables.

**Results:**

We found that feeding fish fat from dietary sources at least once a week during puppyhood was inversely associated with epilepsy in later life in the unadjusted analysis [OR 0.46 (95% CI 0.25–0.83), *p*=0.01], while when adjusting for keeping conditions and dog characteristics the association was [OR 0.45 (95% CI 0.23–0.88), *p*=0.02]. When adjusted for keeping conditions, dog characteristics, and other feeding factors, the association was of similar magnitude but not significance [OR 0.56 (95% CI 0.27–1.15), *p*=0.12].

**Discussion:**

The study indicates possible protective associations of feeding the dog with dietary sources of fish fat against epilepsy, although the result could be confounded by other feeding factors. Findings are compatible with current knowledge regarding the role of omega-3 fatty acids and ketogenic diet, a low carbohydrate, high fat diet as supportive treatments of epilepsy. As our findings are based on observations, we suggest the possibility of causality but do not prove it. Dietary intervention studies should now be conducted to confirm our findings.

## Introduction

Epilepsy is a serious and common neurological condition in both humans and dogs. Canine epilepsy exhibits a striking similarity to human epilepsy including prevalence, etiology, disease course, clinical manifestations, therapeutic response, genetic background, and comorbidities of epilepsy (1, 2). The overall prevalence of epilepsy in dogs is estimated to be from 0.6 to 0.75% (3, 4). A similar epilepsy prevalence ratio can be found in humans (5). The medications used for dogs are also similar to human’s medications such as phenobarbital, potassium bromide, gabapentin, diazepam, and other different human epilepsy drugs used for dogs off-label (6). Canine epilepsy is thought to be multifactorial, including environmental, developmental, and genetic factors (7), and similar issues can be seen in human epilepsy (8). Thus, canine epilepsy provides an ideal model for translational studies (2).

Risk/predisposing factors for epilepsy have been extensively studied in human (9–12) and canine studies (3, 13–15). As an example of early life risk factors, early life stress has been studied in human epilepsy (16, 17). Recent rodent research showed that early life nutrition, among other factors, had a role in mediating the risk of “absence seizure” epilepsy and its comorbidities in WAS/Rij rats (18). Early life nutrition also mediated other neurodegenerative diseases via genetic and epigenetic signatures such as DNA methylation and histone modifications (19).

Nutrition is also used as an alternative treatment in the management of refractory epilepsy which has been found to be uncontrollable with only medications (20–22). High-fat, low-carbohydrate diets are an old well-established and studied form of treating epilepsy in humans (23–29) and nowadays also in dogs (30, 31). Similarly, a medium chain triglyceride enriched diet has been shown to improve seizure control in dogs with epilepsy (32, 33). These diets, also referred to as the classic high-fat ketogenic diets (KDs) and the medium-chain triglyceride KDs, are tailored to induce ketosis, even if the mechanisms of action for seizure control are still disputed (34–36). Brain mitochondrial impairment and/or lower levels of intracellular neuronal oxidative glucose metabolism seem to play a role (37). Impaired glycolysis has many implications, the major one is a depletion of energy availability in the form of ATP (37). The neuronal action potential-based signal transduction is a highly energy-intensive process, especially the stabilization of the membrane potential post-firing, meaning stopping the signal (38). Hence, one theory is that it is the availability of ATP for the sodium-potassium ATPase pump of the neuronal membrane at specific epileptogenic *foci* that determines whether a seizure will occur or not (39). Increasing dietary fatty acid consumption may allow for the liver to produce alternative fuel sources for the brain, i.e., ketone bodies, which can enter the TCA cycle as acetyl-CoA and make ATP instead of glucose (40). Ketone bodies have also been shown to exhibit anticonvulsant properties in the brain (41). We have found only one study that compared ketosis in man and dogs. This last century paper finds no fundamental difference in ketosis in man and dog, although dogs take longer to get into ketosis and their circulating ketone values remain lower (42). Also, only adding omega-3 polyunsaturated fatty acids (n-3 PUFA) to a non-KD ameliorates the neuronal membrane plasticity and its ability to support normal glucose transport to the brain from plasma via the GLUT1 transporter (43, 44). This may also help neurons produce sufficient ATP to control signal transduction via the sodium potassium ATPase membrane pump (45). Other authors have found that epilepsy can be diminished prospectively in mice using KDs (46, 47). Another potential pathway of the role of KDs in epilepsy patients is through the microbiota-gut-brain axis (48), wherein human and canine studies a difference in the microbial composition has been found between epileptic cases and healthy controls (48). A murine study has demonstrated that the gut microbiota can mediate the antiseizure effect of the KD by modulating the hippocampal metabolome which, in turn, correlates with seizures reduction (49).

To our best knowledge, there are no previous reports on the effect of early life nutrition on the later onset of epilepsy in dogs. Our study aims to determine whether dietary fat sources in domestic dogs’ diets during puppyhood are associated with the risk of epilepsy. Accordingly, early life dietary intervention studies should be conducted to shed light on the etiology of epilepsy. As both human and canine epilepsy occur spontaneously and show very similar disease symptoms, we suggest that the results of our canine epilepsy study have also potential transitional benefits.

## Materials and methods

### Study design and the validated DogRisk food frequency questionnaire (FFQ)

As a study design, we used a nested case–control setting with up to four controls per case, matched for the key confounding factors sex, breed, and age. The study material consisted of Finnish companion dogs, whose owners had answered the internet-based DogRisk FFQ. The FFQ was launched in December 2009. The FFQ was open to all Finnish dog owners. The recruitment process has been previously described (50) but will be revisited here shortly. Dog owners were recruited using announcements in public media, dog shows, dog associations, dog food distributors, and veterinary clinics throughout the country. The DogRisk FFQ collected information on 117 disease conditions, including age at first occurrence, recurrence, and in some diseases, also specific diagnosis. As in the FFQ, there is no certainty that the diagnoses were made by veterinarians, therefore dogs with recurrent seizures are termed simply as “epilepsy” throughout the paper. The DogRisk FFQ collected feeding information from three different age groups: Puppyhood, youth and adult. Also, it collected data on the dog’s characteristics, keeping conditions, and exercise habits. The performance of the FFQ in measuring descriptive data and three disease variables has been assessed in a previous validation study (51). Moreover, the FFQ food groups have also been assessed (52).

The data version used in the present analyses was extracted from the web in May 2015 and included a total of 10,030 answers. Survey piloting and robot answers were excluded.

The collection of the initial DogRisk data did not need ethical approval since no sensitive data and no biological samples were collected. However, due to stricter data management regulations nowadays, an ethical approval from the Ethics committee at the Viikki campus, University of Helsinki was applied and granted retrospectively (29.4.2016).

### Study subjects and their characteristics

**Figure 1:**
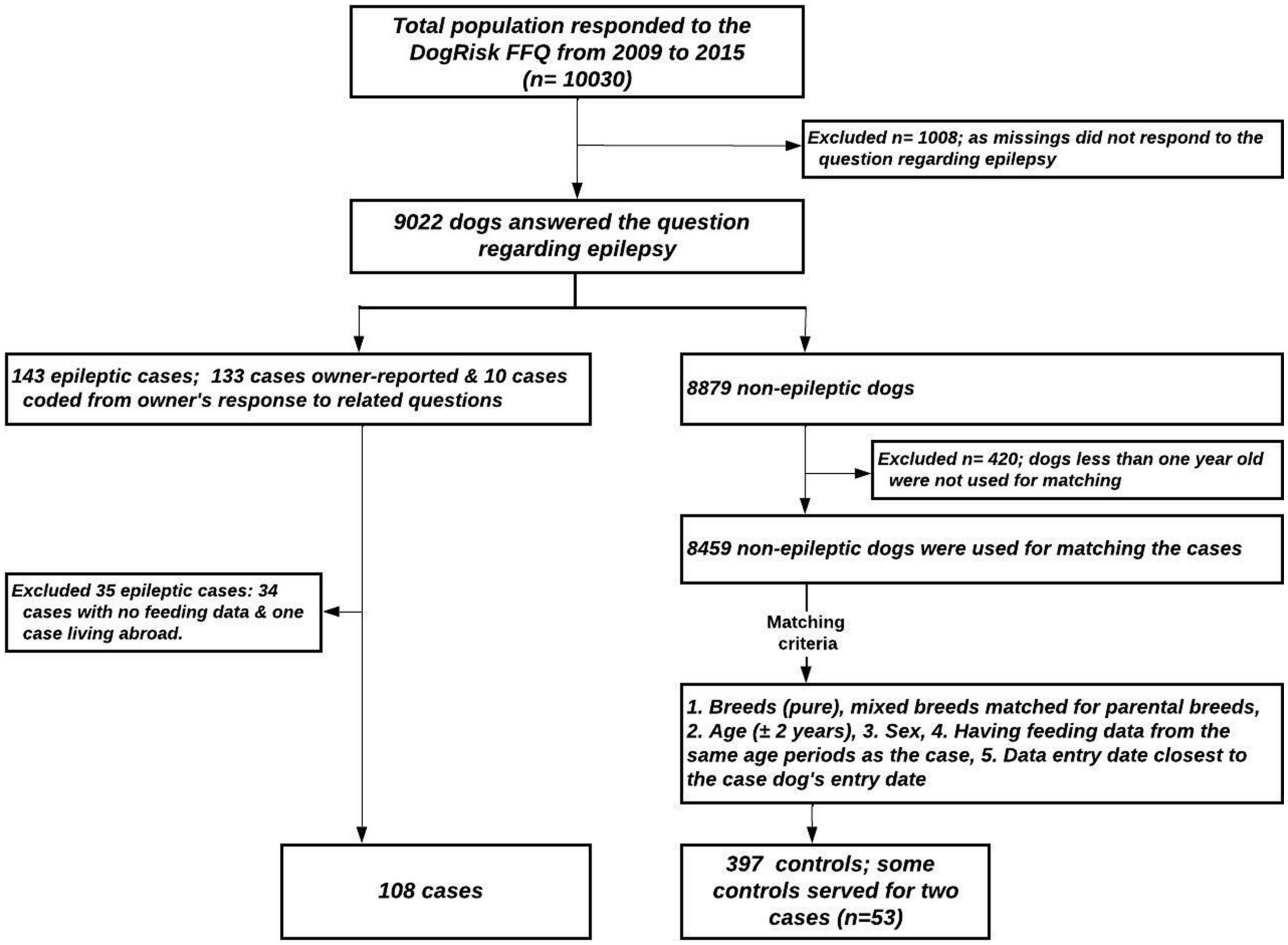
The study flow chart.

In this study, we only looked at diets that the dogs had eaten as a puppy, between 2 and 6 months old. This was to avoid reverse causality, as canine epilepsy can start at a young age (1 year). We calculated a sum variable on energy-containing feeds for each dog. If the dog did not get energy-containing feeds (=proteins, fats and carbohydrates) at least 5 times per week, the feeding information was considered incomplete, and the dog’s data was excluded from the analyses. We identified 108 epileptic dogs from the DogRisk dataset, for each we matched two to four controls from the dataset, which resulted in 397 controls. The controls were matched for breed, sex, and age (± 2 years). The breeds accepted as controls for cases are given as Supplementary Table S1. The study sample inclusion and exclusion criteria are shown in the study flowchart (Figure 1). The same dogs were allowed to serve as controls for two or more cases, so the study sample had fewer dogs (*n* = 452) than the sum of cases and controls (*n* = 505).

For testing the reproducibility of the study estimates, we invited the dog owners of both cases and controls to complete a second online Epilepsy Ascertainment Questionnaire (EAQ) to ascertain the disease information by verifying that the diagnosis of epilepsy was made by a veterinarian, and to collect further information about the disease (data not showed and used here). We were able to contact owners of 400 dogs (88.5%), 258 (57.1%) of which completed the second questionnaire.

### Data curation

The feeding questionnaire had 54 questions, many with drop-down boxes, about the frequency of feeding different food items to the dog. These questions were aimed to cover the total diet. We re-grouped the original feeding variables into broader food groups with similar nutrition content and handling procedure, which then resulted in 55 food variables. These were then combined into 19 major food groups (such as milk-products, meats, fruits and vegetables etc.). All of the original questions had five frequency options ranging from “never” to “daily or almost daily.” The frequencies of feeding variables were transformed into feeding frequency per week. After that, missing values were imputed with null.

No information on the quantity of the foods eaten by the dog was collected but a ratio (in %) of the type of diet that the dog was served, was given. The sum had to equal 100%: How many % of their dog’s diet at puppy-age was (1) ultra-processed dry food, (2) ultra-processed wet/moist food, (3) home cooked food or (4) raw (=not heat-treated) food? These different types of diets already indicate their macronutrient ratios, as, e.g., the extrusion process when making dry food requires a lot of carbohydrates, the moist foods sold in Northern Europe are very similar to the dry foods vis a vis ingredients except water content, whereas raw foods are sold either as raw meats, raw organ meats, raw meaty bones, non-heat treated vegetables, berries and fruits that the owner mixes, or as raw dog food companies’ complete and balanced frozen foods. Either way, the carbohydrate content in raw foods is low, 0–5%, and thereby primarily include animal proteins, fats, fiber, and minerals. The home prepared food is more varied.

The feeding variables of interest in the present study were the main supplementary sources of fatty acids in the dogs’ diet. We analyzed fats in categories of supplemented fish oil (such as salmon oil, other fish oils, cod liver oil etc., summed from three places in the FFQ), supplemented other animal fat (such as meaty lamb, pork or beef fat, butter, lard, tallow etc.), supplemented vegetable oils (30 different including olive, corn, safflower, canola, flaxseed, hemp, coconut etc., also from three places in the questionnaire), and supplemented mixed oils (commercial mixtures of fish and vegetable oils). As we could already see early in our analyses that fish oils were interesting, we also summed the four fish variables that were present in the questionnaire into one variable called “fish” (raw, cooked, dried or where production method was not known) in the analysis, as fish is an important source of long-chain omega-3 fatty acids. Finally, we summed the fish, fish oil, and mixed oil variables to a combined “total fish fat sources” variable. We visually inspected the distribution of each fat category variable. All the variables had a skewed distribution with a high number of non-users. The median value tended to be close to a feeding frequency of once a week. We, therefore, transformed the fat feeding variables into dichotomous “less than once a week” versus “at least once a week” variables for the analyses. Other major food groups were analyzed as potential confounding factors.

### Statistical methods

Statistical analyses were done using IBM SPSS Statistics 22/28. The repeatability of owner-reported epilepsy was assessed by comparing the owner’s responses to the question of whether the dog has epilepsy in the original DogRisk FFQ and the second EAQ, sent later to the chosen owners. We used Cohen’s kappa as the test statistic. The consistency of feeding during the separate age periods (puppy and young) was also tested with Cohen’s kappa statistic.

The supplemented fat variables of interest (fish, fish oil, mixes of fish and vegetable oils, total fish fat sources, other animal fats, and vegetable oil) were tested in three different models, model I for unadjusted analysis, model II for adjusted analysis for keeping conditions and dog characteristics, and model III for adjusted analysis for keeping conditions, dog characteristics, and other feeding factors. Because the number of matched control dogs per each case dog varied, it wasn’t possible to use a standard logistic regression model. We analyzed the association between feeding factors and the risk of epilepsy using the Cox regression model (as described in IBM Support: Conditional logistic regression using COXREG) (53–56). The dependent (time) variables were set to 1 for cases and 2 for controls, and the status variable to 1 for cases and 0 for controls. We used the code denoting each case–control group as the paired variable.

To select the background variables to be used in the statistical model as potential confounding factors, we tested the associations of 38 different variables concerning the characteristics of the dog and its circumstances with epilepsy in adulthood using the univariate Cox regression model in a similar manner as in the feeding analyses. The variables with a *p*-value of ≤0.20 and *n* ≥ 380 were included simultaneously in a Cox regression model. Variables that had a *p*-value of ≤0.20 in model II & III (age, season of birth, other dogs in the household, dewormed as a puppy, dewormed at least twice a year, kept free daily, daily hours kept free on a yard, never kept unleashed, almost always unleashed) were used as adjusting factors in models analyzing the associations of fat feeding factors and epilepsy later in life.

Similarly, we tested the associations of 19 other feeding factors, to be used in the statistical model as potential confounding factors, with epilepsy in adulthood using the univariate Cox regression model and added the variables with a *p*-value of ≤0.20 simultaneously in the model including also the selected background factors. Variables that had a *p*-value of ≤0.20 in model III (feeding fruits or vegetables, and moist ultra-processed dog foods at least once a week as a puppy) were used as adjusting factors in models analyzing the associations of fat feeding factors and epilepsy later in life.

The ratio of the four major diets (dry, moist, home-made, or raw) were reorganized into five ordinal groups: 0%, 1–25%, 26–50%, 51–75%, and 76–100% of the different diets. All four diets were tested separately against epilepsy or not in adulthood, using crosstabulation.

The statistical analyses were performed using two separate endpoint variables with differing levels of information available and thus different certainty of the diagnosis. The first endpoint “Owner-reported epilepsy” is based on the response of the owner on the original DogRisk FFQ, corrected by the owner’s response in the EAQ in cases of contradictory information between the two questionnaires.

As the second endpoint, we used the owner’s statement that the dog’s epilepsy has been diagnosed by a veterinarian. Because this information was asked only in the EAQ, and not in the original DogRisk FFQ, the case numbers are smaller than in the first endpoint even if most epilepsies were in fact diagnosed by a veterinarian.

## Results

The breed distribution of case and control dogs is shown in Supplementary Table S2. The mean age of cases and controls was 7.2 years and 6.5 years, respectively. The median age of both cases and controls was 6 years and 54% of both cases and controls were females.

We assessed the repeatability of the owner’s response about the dog’s epilepsy status by inviting the epileptic cases and their matched controls to complete an EAQ. We also tested the agreement between these repeated questions using Cohen’s kappa statistic. The kappa value was 0.88 (*n* = 243, *p* < 0.001). The median time between completing the two questionnaires was 65 months (range 15–80 months). The agreement between the owner’s answer to the question whether the dog has epilepsy on the original DogRisk FFQ, and the question whether the dog has epilepsy diagnosed by a veterinarian on the EAQ, was 0.69 (*p* < 0.001), calculated using the Cohen’s kappa statistic.

We also analyzed the degree of consistence in feeding habits between puppyhood and young age using Cohen’s kappa statistic (Supplementary Table S3). Using dichotomous variables of feeding at least once a week versus less often, the agreement between puppy and young dog age periods was high for all the feeding variables. We found a high degree of continuity in feeding habits across age periods. Dietary fat feeding was especially stable between the puppyhood and youth periods. The agreement was over 90% in all analyzed variables.

The ratio analyses of dogs eating from the four separate diet styles showed no significant association with having epilepsy in adulthood or not (all *p*-values >0.234).

**Figure 2:**
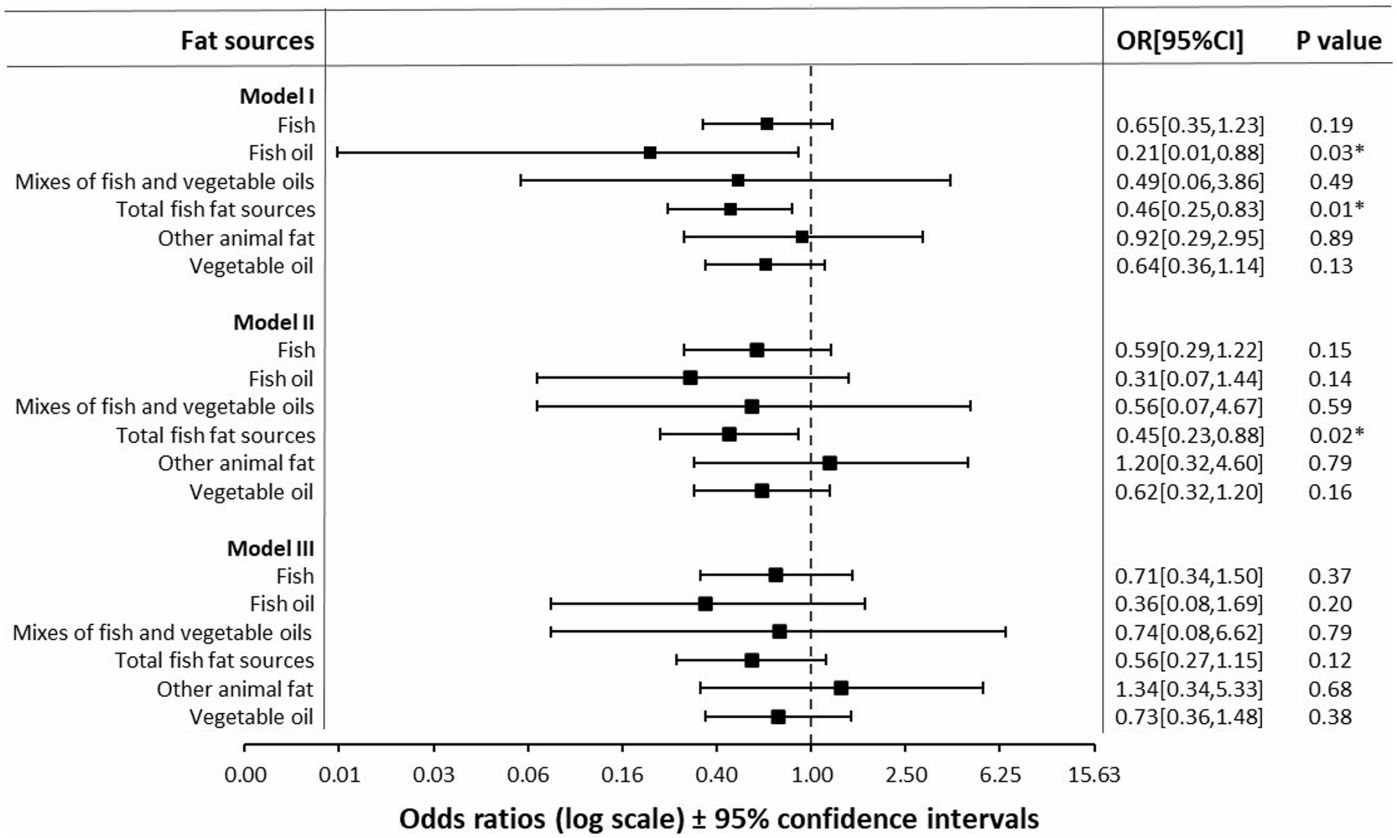
Odds ratios of the associations between puppyhood feeding from 2 to 6 months of age with fat sources and the risk of owner-reported epilepsy in later life in a nested case–control study among Finnish companion dogs, analyzed using Cox regression model. **Model I** for Unadjusted analysis with *n* = 398, 86 cases, **model II** for adjusted analysis for keeping conditions and dog characteristics (age, season of birth, other dogs in the household, dewormed as a puppy, dewormed at least twice a year, kept free on a yard daily, daily hours kept free on a yard, never kept unleashed, almost always unleashed) with *n* = 374, 84 cases, and **model III** for adjusted analysis for keeping conditions and dog characteristics (age, season of birth, other dogs in the household, dewormed as a puppy, dewormed at least twice a year, kept free on a yard daily, daily hours kept free on a yard, never kept unleashed, almost always unleashed), and other feeding factors (feeding fruits or vegetables, and moist ultra-processed dog foods at least once a week as a puppy) with *n* = 374, 84 cases.

Fish oil and total fish fat sources given at least once a week during puppyhood were inversely associated with the risk of epilepsy in later life in the unadjusted analyses (Figure 2). For total fish fat sources, the association was significant when adjusted for keeping conditions and dog characteristics. None of the analyzed puppyhood fat feeding variables were significantly associated with the risk of epilepsy when adjusted for background and feeding factors, although the odds ratios for fish oil and total fish fat sources were still small and compatible with a protective association (Figure 2). None of the confounding variables included in the analyses remained significant in any of the final models.

The results for epilepsy diagnosed by a veterinarian with confirmed certainty (second endpoint) were similar, although the case numbers were smaller and statistically significant results were observed less often. The results are presented in the Supplementary Table S4.

## Discussion

The findings from the current epidemiological analysis in a population of Finnish companion dogs indicated a significant negative association of feeding the dog dietary sources of fish fat during puppyhood with the incidence of epilepsy, although other feeding factors may play a role.

We analyzed the feeding frequencies of dietary fats and oils in relation to the risk of epilepsy, including fish as it is a typical source of long-chain omega-3 fatty acids. To better discern the possible associations of fish-derived fat with the risk of epilepsy, we calculated a sum variable that combined the different sources of fish fat in the dogs’ diet. Feeding with different dietary sources of fish fat during puppyhood was inversely associated with the risk of epilepsy with all the odds ratios being on the protective side (<1). The protective association was statistically significant in the unadjusted analysis and in the analysis adjusted for keeping conditions and dog characteristics. When the analysis was adjusted for these and additionally for other feeding factors, we found no significant protective association, which could indicate that other feeding factors also playing a role. These other feeding factors were however tested separately both as combined food item groups (such as milk products, eggs, meats etc.) and as ratios of the four different diet types, and none of these associated with adulthood epilepsy. Feeding with fish oil during puppyhood also showed a significant protective association in the unadjusted analysis. In a previous study looking for dietary risk factors of allergy/atopy skin signs during puppyhood we found that fish oil was significantly associated with a lower incidence of allergy/atopy skin signs when given once per year while giving the fish oil supplement frequently or several times per week was associated significantly with a high risk of allergy/atopy skin signs in dogs (57). We will now put our results into a wider perspective of nutritional fats and epilepsy. As fish fats were more protective than other fats, most of our discussion below will be on omega-3 fatty acids and not on the ketogenic diet.

A review and an owner questionnaire indicate that veterinarians and owners in the UK are aware of the protective effects of fats for epilepsy in dogs (48, 58). Middle chain triglycerides (MCTs) are known to be especially suitable for making ketones but in our data, we only had one control dog that had received coconut oil, the only typical MCT oil we had as an option in a drop-down box for the owners to choose from. Fish fat is a known source for n-3 PUFA, docosahexaenoic acid (DHA), and eicosapentaenoic acid (EPA). One study reported that mice fed a diet deficient in n-3 PUFA for 1 month were more predisposed to audiogenic seizures than those fed a diet with adequate n-3 PUFA (59). Another study found that maternal n-3 PUFA supplementation in seizure-resistant rat strains greatly reduced their seizure sensitivity whereas it was increased in a seizure-prone rat strain (60). Polyunsaturated fatty acids have shown protective effects against artificially induced seizures in experimental animals (61–64). In most of these studies, fatty acids were supplied as injections (62–64), so the metabolism may differ from orally supplied ones (23). Linoleic acid has been most often used (61–64), sometimes in combination with alpha-linolenic acid (61, 62). In one of the studies, the seizure protective effects of EPA and DHA were greater than those of linoleic and oleic acids (64). These findings agree with the stated hypothesis that the consumption of polyunsaturated fatty acids during early life plays a protective role against epileptic seizures.

There are only a few clinical studies on fatty acids in the management of canine epilepsy, and with few study subjects: According to a case report, a reduction of seizure frequency was seen in a dog with drug-resistant epilepsy after supplementation with fish oil (65). On the contrary, in a small, blinded, placebo-controlled trial among dogs with idiopathic epilepsy, a 12-week supplementation with a mixture of EPA, DHA, and vitamin E failed to reduce the severity or frequency of seizures (66). Among humans, supplementation with polyunsaturated fatty acids was linked to reduced seizure frequency but the study had only 5 patients and no control group (67). In a small, randomized placebo-controlled human study, a lower dose of two fish oil was associated with a reduction in seizure frequency, whereas the higher dose had no effect when compared to the placebo treatment with corn oil (68). Similarly, in a pilot trial from the same study group, a high dose of fish oil tended to increase seizure frequency (69). In another randomized, placebo-controlled trial, n-3 fatty acid supplementation reduced seizure frequency during the first 6 weeks of treatment (70). Interestingly, the authors speculated whether this reduction was due to the previously reported short-time rise in blood concentration of arachidonic acid during supplementation with low doses of EPA and DHA. If this is the case, then lower doses of n-3 fatty acids could be more effective than higher doses in the treatment of epilepsy, as suggested in schizophrenia and depression (71). The dose–response curves of n-3 fatty acids with many endpoints may be bell-shaped, and the optimal health effects may require a balanced supply of both n-6 and n-3 fatty acids (71). Therefore, the benefits observed in our results from eating fish may be explained by the balance of n’6 and n’3 fatty acids when consumed as a food.

The success of omega-3 fats in the diet (or by using a ketogenic high fat diet) in the reduction of seizure frequency in humans and dogs as discussed above, indicates that the seizure disorder might be based on irregularities in the fatty acid metabolism, although not all studies have given similar results (72). Deficiency of fatty acids has also been reported to be associated with the development of seizure disorder, autistic spectrum disorder, and attention deficit hyperactivity disorder (60). Deficiency of essential fatty acids, especially DHA, during the developmental periods associates with different types of epilepsy (73), which indicates that the proper DHA supplements are essential for optimal neurodevelopment. Deficiency of fatty acids during the pre- and early postnatal life periods has been associated with a delay in the growth and maturation of the myelin in the brain frontal lobe of offspring (74). In seizure disorder, n-3 PUFA supplementation has been found to promote neuroprotection (75). The n-3 PUFA can play a vital role in neuronal functions throughout the life via several proposed mechanisms (76) including maintaining the cell membrane liquidity (77), myelination (78), neural growth, gene expression (76), and its anti-inflammatory properties (79). The changes in the cell membrane liquidity due to the supplementation of essential fatty acids are associated with neurotransmission, ion channel regulation, and gene expression. As inflammation has also been suggested to be involved in the pathophysiology of canine epilepsy (80) and as n-3 PUFAs have anti-inflammatory properties (81) which increase when exposed to inflammatory challenges (82) this is an additional possible success factor.

### Strengths and limitations

To summarize, the main strengths of the present study are the large sample size with several breeds, the matched case–control setting, several different type of confounding factors, and the detailed food frequency information. Moreover, the longitudinal setup of the data could have allowed for exploring the food frequency also during young age, but as epilepsy can erupt already at an early age, we did not look at the feeding of young dogs in this study, but we tested how consistent the diet still was in the adolescent dog. The study also tested two cohorts, “owner-reported” and “veterinarian diagnosed epilepsy,” with a high consistency rate. It is also still possible that the association between fish intake and epilepsy is explained by some other feeding factors, or by the combined feeding styles, but as a strength we can say that we tried to control for these and that we also tested the diet ratios separately. The major limitations are the lack of information on feeding amounts, and the possible recall bias, owners of epileptic dogs may have remembered the dog’s feeding differently than the healthy dogs’ owners. For instance, if they have thought a lot about the dog’s health and its feeding, they may have reported the diet in greater detail.

## Conclusion

In conclusion, this is the first epidemiologic study on the associations between dietary fat intake during puppyhood and the risk of epilepsy, conducted in a study population of Finnish companion dogs. The study suggested a possible protective association of feeding puppy aged dogs with dietary sources of fish fat against epilepsy later in life. The results are compatible both with earlier observations of long chained omega-3 fatty acids that are found in fish as a treatment of epilepsy, and they have also a theoretical support concerning the seizures’ potential mechanism of action. The effects of different feeding factors cannot be reliably separated in an observation study because they tend to be intertwined. Furthermore, a single epidemiological study is always only indicative. Therefore, a randomized, controlled longitudinal dietary intervention with dietary fatty acids in the prevention of epilepsy is required to confirm our results.

## Data availability statement

The raw data supporting the conclusions of this article will be made available by the authors, without undue reservation.

## Ethics statement

Ethical approval from the Ethics Committee at the Viikki campus, University of Helsinki was applied and granted retrospectively (29.4.2016).

## Author contributions

MH and AH-B planned, designed, drafted the study, performed the data extraction and did the statistical analysis, tables, and figures. AH-B organized the database. MH, SR, TJ, RM, KV, JA, and AH-B wrote sections of the manuscript and edited it. All authors contributed to manuscript revision, read, and approved the submitted version.

## Supplementary material

SUPPLEMENTARY TABLE S1 Breeds accepted as controls for epileptic cases with insufficient number of eligible controls before the substitution.

SUPPLEMENTARY TABLE S2 Breed distribution of 108 dogs with epilepsy and 397 non-epileptic control dogs matched for breed, age and sex.

SUPPLEMENTARY TABLE S3 Consistence of feeding habits between the puppyhood and young age among Finnish companion dogs that became epileptic (*n*=108) and non-epileptic control dogs (*n*=397).

SUPPLEMENTARY TABLE S4 Associations between puppyhood feeding with fat sources and the risk of epilepsy diagnosed by veterinarian in later life in a nested case-control study among Finnish companion dogs, analyzed using Cox regression model. Separate model for each cell of the table.

## Abbreviations

FFQ: food frequency questionnaire
n-3 PUFA: omega-3 polyunsaturated fatty acids
DHA: docosahexaenic acid
EPA: eicosapentaenic acid
KD: ketogenic diet
