# Supplementary material for: Assessing the association between supplemented puppyhood dietary fat sources and owner-reported epilepsy in adulthood, among Finnish companion dogs
Source: Front Vet Sci. 2023 Sep 15;10:1227437. doi: 10.3389/fvets.2023.1227437 (PMC10540444; doi:10.3389/fvets.2023.1227437)
Supplement: SUPPLEMENTARY TABLE S1 — Breeds accepted as controls for epileptic cases with insufficient number of eligible controls before the substitution. [file Table_1.DOCX]

Table S1. Breeds accepted as controls for epileptic cases with insufficient number of eligible controls before the substitution.

| Breed of the case dog | Breeds accepted as control dogs |
| --- | --- |
| Toy poodle | Miniature poodle |
| English springer spaniel | Other British spaniel breeds: cocker spaniel, field spaniel, clumber spaniel, sussex spaniel |
| Basset hound | Basset fauve de Bretagne, Basset bleu de Gascogne, Basset artesien Normand, Petit basset griffon Vendeen, Grand basset griffon Vendeen |
| Cao da Serra de Aires | Pyrenean mountain dog, Catalan sheepdog, Briard |
| German wire-haired pointing dog | German short-haired pointing dog, Deutsch langhaar |
| Long-haired standard dachshund | Other standard dachshund varieties: Smooth-haired, wire-haired |
| Italian volpino | German spitz: Pomeranian, miniature spitz, medium size spitz |
| Boston terrier | Bulldog, French bulldog |
| Japanese spitz | Medium size spitz |
| Norwich terrier | Cairn terrier, Scottish terrier, Sealyham terrier, West highland white terrier |
| Belgian shepherd dog tervueren | Other Belgian shepherd dog varieties: laekenois, malinois, groenendael |
| Giant schnauzer | Schnauzer |
