# Supplementary material for: Assessing the association between supplemented puppyhood dietary fat sources and owner-reported epilepsy in adulthood, among Finnish companion dogs
Source: Front Vet Sci. 2023 Sep 15;10:1227437. doi: 10.3389/fvets.2023.1227437 (PMC10540444; doi:10.3389/fvets.2023.1227437)
Supplement: SUPPLEMENTARY TABLE S2 — Breed distribution of 108 dogs with epilepsy and 397 non-epileptic control dogs matched for breed, age and sex. [file Table_2.DOCX]

Table S2. Breed distribution of 108 dogs with epilepsy and 397 non-epileptic control dogs matched for breed, age and sex.

|  | Cases | Controls |
| --- | --- | --- |
| Breed |  |  |
| Airedale terrier | 0.9 % (1) | 1.0 % (4) |
| Australian shepherd | 1.9 % (2) | 1.8 % (7) |
| Basenji | 0.9 % (1) | 1.0 % (4) |
| Grand basset griffon vendeen | 0.9 % (1) |  |
| Basset hound | 1.9 % (2) | 0.8 % (3) |
| Basset artesien Normand |  | 0.3 % (1) |
| Basset fauve de Bretagne |  | 0.3 % (1) |
| Grand basset griffon Vendeen |  | 0.5 % (2) |
| Petit basset griffon Vendeen |  | 0.3 % (1) |
| Belgian shepherd dog, groenendael | 1.9 % (2) | 2.3 % (9) |
| Belgian shepherd dog, laekenois |  | 0.3 % (1) |
| Belgian shepherd dog, malinois | 0.9 % (1) | 1.0 % (4) |
| Belgian shepherd dog, tervueren | 1.9 % (2) | 0.8 % (3) |
| Bernese mountain dog |  | 1.0 % (4) |
| Boxer | 1.9 % (2) | 2.0 % (8) |
| Border collie | 3.7 % (4) | 4.5 % (18) |
| Border terrier | 2.8 % (3) | 3.0 (12) |
| Boston terrier | 0.9 % (1) |  |
| Brazilian terrier | 1.9 % (2) | 2.0 % (8) |
| Briard |  | 0.8 % (3) |
| Cavalier King Charles spaniel | 1.9 % (2) | 1.5 % (6) |
| Chihuahua | 0.9 % (1) | 1.0 % (4) |
| English springer spaniel | 1.9 % (2) | 1.3 % (5) |
| Bulldog |  | 0.3 % (1) |
| Field spaniel | 0.9 % (1) | 0.8 % (3) |
| Jack Russell terrier | 1.9 % (2) | 1.5 % (6) |
| Japanese spitz | 1.9 % (2) | 0.3 % (1) |
| Curly coated retriever | 0.9 % (1) | 1.0 % (4) |
| Golden retriever | 2.8 % (3) | 3.0 (12) |
| Miniature pinscher | 1.9 % (2) | 2.0 % (8) |
| Miniature spitz |  | 0.3 % (1) |
| Miniature poodle | 2.8 % (3) | 3.8 % (15) |
| Labrador retriever | 5.6 % (6) | 6.5 % (26) |
| Finnish Lapphund | 2.8 % (3) | 4.1 % (16) |
| Medium size spitz | 0.9 % (1) | 3.0 (12) |
| Mixed breed | 21.3 (23) | 16.1 % (64) |
| Pug | 4.6 % (5) | 4.8 % (19) |
| Dachshund | 2.8 % (3) | 4.1 % (16) |
| Norwegian elkhound grey | 0.9 % (1) | 0.5 % (2) |
| Norwich terrier | 0.9 % (1) |  |
| Nova Scotia duck tolling retriever | 2.8 % (3) | 3.0 (12) |
| Parson Russell terrier | 1.9 % (2) | 1.5 % (6) |
| Bearded collie | 0.9 % (1) | 1.0 % (4) |
| German pinscher | 0.9 % (1) | 1.0 % (4) |
| Pumi | 0.9 % (1) | 0.5 % (2) |
| Pyrenean mountain dog |  | 0.3 % (1) |
| Pyrenean sheepdog |  | 0.5 % (2) |
| Miniature spitz | 0.9 % (1) | 1.5 % (6) |
| German wire-haired pointing dog | 0.9 % (1) |  |
| German short-haired pointing dog | 0.9 % (1) | 1.3 % (5) |
| Cao da Serra de Aires | 0.9 % (1) |  |
| Schnauzer | 0.9 % (1) | 2.0 % (8) |
| Staffordshire bull terrier | 0.9 % (1) | 1.0 % (4) |
| Finnish spitz | 1.9 % (2) | 2.3 % (9) |
| Giant schnauzer | 1.9 % (2) | 1.0 % (4) |
| Toy poodle | 0.9 % (1) | 0.3 % (1) |
| West Highland white terrier | 0.9 % (1) | 1.3 % (5) |
| Italian volpino | 0.9 % (1) |  |
| Welsh springer spaniel |  | 0.3 % (1) |
| Welsh corgi cardigan | 0.9 % (1) | 1.0 % (4) |
