# Supplementary material for: Assessing the association between supplemented puppyhood dietary fat sources and owner-reported epilepsy in adulthood, among Finnish companion dogs
Source: Front Vet Sci. 2023 Sep 15;10:1227437. doi: 10.3389/fvets.2023.1227437 (PMC10540444; doi:10.3389/fvets.2023.1227437)
Supplement: SUPPLEMENTARY TABLE S3 — Consistence of feeding habits between the puppyhood and young age among Finnish companion dogs that became epileptic (n=108) and non-epileptic control dogs (n=397). [file Table_3.DOCX]

Table S3. Consistence of feeding habits between the puppyhood and young age among Finnish companion dogs that became epileptic (n=108) and non-epileptic control dogs (n=397).

|  | Percentage of dogs in the same category at both age periods  (Cohen’s kappa statistic) |
| --- | --- |
| Feeding frequency:  at least once a week vs less often | **Puppy^1^ vs young^2^** |
| Fish | 95.0 (0.84) |
| Fish oil | 99.0 (0.94) |
| Mixes of fish and vegetable oils | **98.0 (0.63)** |
| Total fish fat sources | 94.2 (0.86) |
| Other animal fat | **99.0 (0.88)** |
| Vegetable oil | 90.2 (0.78) |

^1^ 2-6 months of age

**^2^** 6-18 months of age
