# Supplementary material for: Assessing the association between supplemented puppyhood dietary fat sources and owner-reported epilepsy in adulthood, among Finnish companion dogs
Source: Front Vet Sci. 2023 Sep 15;10:1227437. doi: 10.3389/fvets.2023.1227437 (PMC10540444; doi:10.3389/fvets.2023.1227437)
Supplement: SUPPLEMENTARY TABLE S4 — Associations between puppyhood feeding with fat sources and the risk of epilepsy diagnosed by veterinarian in later life in a nested case-control study among Finnish companion dogs, analyzed using Cox regression model. Separate model for each cell of the table. [file Table_4.DOCX]

Table S4. Associations between puppyhood^1^ feeding with fat sources and the risk of epilepsy diagnosed by veterinarian in later life in a nested case-control study among Finnish companion dogs, analyzed using Cox regression model. Separate model for each cell of the table.

|  | OR, 95 % CI, *p-*value | | |
| --- | --- | --- | --- |
| Feeding frequency:  at least once a week vs less often | **Unadjusted**  n=148, 32 cases | **Adjusted for keeping conditions and dog characteristics^2^**  n=148, 32 cases | **Adjusted for keeping conditions, dog characteristics, and other feeding factors^3^**  n=148, 32 cases |
| Fish | 0.90 (0.34-2.40), *0.84* | 0.79 (0.26-2.39), *0.68* | 0.88 (0.28-2.74), *0.83* |
| Fish oil | 0.19 (0.02-1.50), *0.12* | 0.21 (0.03-1.70), *0.14* | 0.26 (0.03-2.09), *0.21* |
| Mixes of fish and vegetable oils | 0.94 (0.11-8.44), *0.96* | 1.11 (0.11-11.2), *0.93* | 1.08 (0.10-12.1), *0.95* |
| Total fish fat sources | 0.39 (0.16-0.98), *0.05* | 0.34 (0.13-0.94), *0.04* | 0.39 (0.14-1.11), *0.08* |
| Other animal fat | 1.41 (0.25-7.93), *0.70* | 1.41 (0.23-8.60), *0.71* | 2.44 (0.38-15.6), *0.35* |
| Vegetable oil | 0.69 (0.28-1.68), *0.41* | 0.72 (0.28-1.82), *0.48* | 0.78 (0.30-2.09), *0.63* |

^1^ 2-6 months of age

^2^ Other dogs in the household, kept almost always unleashed.

^3^ Feeding human foods and leftovers at least once a week as a puppy
